# Familiarity influences on proactive interference in verbal memory

**DOI:** 10.1177/17470218251317191

**Published:** 2025-02-14

**Authors:** Tom Mercer

**Affiliations:** Centre for Psychological Research, School of Psychology, University of Wolverhampton, Wolverhampton, UK

**Keywords:** Proactive interference, verbal memory, familiarity, recent-probes task, time

## Abstract

Proactive interference occurs when older memories interfere with current information processing and retrieval. It is often explained with reference to familiarity, where the reappearance of highly familiar items from the recent past produces more disruption than older, less familiar items. However, there are other forms of familiarity beyond recency that may be important, and these were explored in a verbal recent-probes task. Participants viewed eight targets per trial and then determined whether a probe matched any of those targets. Probes matching a target from the previous trial, rather than an earlier trial, led to more errors, revealing proactive interference. However, this effect was influenced by experimental familiarity (whether stimuli were repeated or unique) and pre-experimental familiarity (whether stimuli were meaningful words or meaningless non-words). Specifically, proactive interference was strongest for repeated non-words, and smallest for unique non-words, but stimulus repetition had little impact for words. In addition, the time separating trials (temporal familiarity) was unrelated to proactive interference. The present findings revealed more complex effects of familiarity than have previously been assumed. To understand proactive interference in a working memory task, it is necessary to consider the role of long-term memory via experimental and pre-experimental stimulus familiarity.

Proactive interference (PI) occurs when memories formed in the past disrupt information processing and retrieval in the present (Badre & Wagner, 2005). It is a robust and well-established mechanism (see Jonides & Nee, 2006), part of formal memory theories (Brown et al., 2007) and the focus of much research effort over decades (Baddeley & Scott, 1971; Keppel & Underwood, 1962; Samrani & Persson, 2024). Managing PI may be one of the primary functions of working memory (Engle, 2002), but impairments in achieving this can be seen in older age (Samrani et al., 2017) and in those with short-term memory deficits, where PI is exaggerated (Hamilton & Martin, 2007; Martin & Lesch, 1996).

PI is known to have a robust effect on verbal memory too, as was famously demonstrated by Keppel and Underwood (1962) in the Brown–Peterson task. In this procedure, participants typically encode three verbal items, such as consonants or words, followed by a distractor task that prevents rehearsal over a retention interval. At the end of the interval, retrieval for the three items is tested, with similar trials following each response. Keppel and Underwood found that recall declined over the course of several trials, which was attributed to a buildup of PI. This effect has been extensively replicated, being found for both recall (Craik & Birtwistle, 1971) and recognition (Carey, 1973). Importantly, however, PI can be alleviated by changing the material being remembered. This was first reported by Wickens et al. (1963), who discovered that the performance decline over several trials in the Brown–Peterson task can be reduced by switching the stimuli (digits or consonants) being remembered. This “release-from-PI” has been extensively replicated under different arrangements (Turvey & Fertig, 1970), though changes to the semantic content of the to-be-remembered stimuli may exert the biggest facilitatory effect (Wickens, 1972).

The buildup and release from PI found in the Brown–Peterson task reflects a non-specific form of interference but re-experiencing a previously encoded item during a subsequent recognition task can produce item-specific PI. This has been most clearly demonstrated in Monsell’s (1978) recent-probes task, in which a set of to-be-encoded targets is followed by an individual probe. The PI effect is revealed on trials where a “Recent Negative” (RN) probe, which matches a target from the immediately preceding trial, slows responding and/or increases errors in comparison with non-recent negative (NRN) probes, which usually match an item from much earlier in the experiment (Jonides & Nee, 2006). This RN/NRN difference provides a behavioural index of PI.

Although the PI found in the Brown–Peterson and recent-probes tasks may differ in specificity, both forms of PI can be attributed to retrieval processes. Early debate concerning the locus of PI (Bennett, 1975; Chechile & Butler, 1975; Dillon, 1973; Hawkins et al., 1972; Loftus & Patterson, 1975; Petrusic & Dillon, 1972) was resolved following evidence that PI hinders retrieval. For example, Gardiner et al. (1972) examined the release-from-PI effect in the Brown–Peterson task. Participants encoded and then recalled word trigrams from a specific subcategory (e.g., wildflowers) for three trials, and on the critical fourth trial the words came from another related subcategory (e.g., garden flowers). Gardiner et al. showed that the provision of a cue denoting the specific subcategory to be retrieved led to clear release from PI, whereas this was not the case for a control group that lacked this cue. This indicates that a simple change in material is insufficient to overcome PI, and instead the effect is determined by the availability of retrieval cues. Other evidence is consistent with this notion (Watkins & Watkins, 1975; Wickens et al., 1981) and Tehan and Humphreys (1996) showed that PI occurs if a recall cue prompts both a target and interfering item, but if the cue only prompts the target item, PI is eliminated.

More recently, Öztekin and McElree (2007) were able to show exactly how PI affected familiarity assessments during retrieval in a task adopting elements of both the Brown–Peterson and recent-probes tasks. Eight participants were extensively tested and on each trial were shown six words from a specific semantic category. After a brief masking stimulus, a single probe was displayed and participants indicated if it matched any of those words, though they were cued to respond by an auditory signal presented at different onset periods. PI was manipulated in two ways. First, words presented every three trials were from a specific category (e.g., fruit), and then there was a switch to a new category set (e.g., animals), in line with the release-from-PI effect of Wickens et al. (1963). In addition, negative probes—i.e., a word not matching those in the current set—were selected either from the previous trial (RN) or a much earlier trial (NRN), following the recent-probes task. Data suggested that when retrieval must occur rapidly, as indicated by the auditory cue, PI disrupts a retrieval process that is based on a rapid assessment of familiarity. The presence of PI makes individual item memories less distinctive—especially as they were drawn from the same semantic category. At a later retrieval stage, however, additional episodic information may be used, which is unaffected by PI. For RN probes specifically, Öztekin and McElree suggested that they have a higher level of residual familiarity, due to being studied more recently than NRN probes, which increases false alarms.

The important role of familiarity is also captured by three theories designed to explain item-specific PI in the recent-probes task. The familiarity-inhibition model (see Badre & Wagner, 2005) suggests familiar stimuli are more likely to elicit a matching response, which is useful on positive trials, where the probe does match one of the current targets. RN probes are also highly familiar due to their recent occurrence, but as familiarity is incorrectly associated with a “match” response, this creates a conflict during responding that leads to lower performance (i.e., more errors or slower responding). In this account, PI resolution is about inhibiting the familiarity signal produced by RN probes (Irlbacher et al., 2014).

The alternative context-retrieval model also states that the familiarity signal on RN trials needs to be controlled, but this is realised by using episodic details such as spatial or temporal information (Badre & Wagner, 2005). The use of these details would allow the RN probe to be correctly assigned to the previous rather than current trial, but due to its more recent occurrence, this does not always occur (Moss et al., 2018).

A third account, the biased-competition model (Jonides & Nee, 2006), which is more specific about the familiarity signal, suggests that an attentional template guides responding. This template has information relevant to the procedure and in the recent-probes task, each probe is compared against the attentional template. Increased similarity between the probe and the template makes a “match” response more probable, and this is more likely to incorrectly occur on RN than NRN trials, due to higher probe-template similarity.

In all these accounts, recency familiarity appears critical, as RN items are encoded closer to the present than NRN items. This concept can usefully explain the specific form of PI emerging in the recent-probes task, but it is unclear whether other forms of familiarity can influence this effect and change the magnitude of PI. Even so, the three accounts seem to allow the RN probe to vary in familiarity, which should affect the intensity of PI. For instance, on the basis of the context-retrieval model, a lengthy delay between trials may reduce the negative impact of RN probes, as it should make it easier to assign it to a previous context. The familiarity-inhibition model and biased-competition model may also expect temporal separation trials to decrease familiarity with items from a previous trial and thereby lower PI.

The effect of time on PI is, however, unclear. In the Brown–Peterson task, inserting long delays between trials is generally helpful for lowering PI (Gorfein & Jacobson, 1972; Kincaid & Wickens, 1970; Loess & Waugh, 1967; Peterson & Gentile, 1965; Turvey et al., 1970), but these effects can be complex. Lorsbach (1990) had no delay or a 90 s gap between Brown–Peterson trials and found notably better performance for the third list following the 90 s delay, but this was less evident for earlier lists. Other studies have found that PI can continue to exert an effect even if a delay of minutes separates trials (Hopkins et al., 1972) and in some cases PI only emerges after a brief delay, not being found in an immediate retention test (Humphreys & Tehan, 1992; Tehan & Humphreys, 1995).

Within the verbal recent-probes task, Berman et al. (2009) varied the inter-trial interval across seven experiments but found it had minimal impact on PI. In contrast, Campoy (2012) was able to find some alleviation from PI at very short delays when using digits, but the effect did not disappear entirely. Unfortunately, both studies may have been affected by ceiling performance, which precluded the error rate from being formally assessed. When a small number of verbal targets are used in the recent-probes task, error rates may only be 1%–2% (Jonides et al., 1998) and accuracy above 90% is not uncommon (Zhang et al., 2010). This meant that Berman et al. and Campoy had to focus on response time, and although this can yield a PI effect, it tends to be relatively modest. Across the experiments by Berman et al., RN trials were, on average, 78 ms slower than NRN trials, though this varied between 47 and 116 ms, depending on the experiment and the inter-trial interval. For Campoy, RN trials averaged 56 ms slower than NRN trials (but this ranged between 24 and 133 ms according to the experiment and delay length). As such, there is need to further examine the effect of temporal familiarity in the verbal recent-probes task when ceiling effects are prevented.

It should also be stressed that the general concept of familiarity goes well beyond simple temporal proximity. Examples include the size of the stimulus pool used within the experiment, which can be thought of as a form of experimental familiarity (i.e., how familiar are the items used throughout the experiment), and prior familiarity with stimuli outside the experimental setting. This is referred to as pre-experimental familiarity, following the study by Toth et al. (2022).

In terms of experimental familiarity, extensive repetition of stimuli throughout the procedure may change the PI effect in the recent-probes task. Tays et al. (2009) noted that the use of English letters within a typical recent-probes procedure would mean individual items being presented approximately 70 times in the experiment, and they did find an increase in errors on the recent-probes task when stimuli were selected from a small pool of 20 words, compared to a much larger set of 750 words.

Repetition of stimuli throughout the procedure, producing high experimental familiarity, may increase PI (McElree et al., 1999), but in the wider literature, the effect of stimulus repetition remains unclear. In the Brown–Peterson task, where the buildup of PI can be monitored, repetition of targets from a previous trial tends to have a facilitatory effect (Cermak, 1969, 1970; Cermak & Sampson, 1972; Goggin & Riley, 1974; Hasher et al., 1973; Wickens & Contrucci, 1974; Wickens & Gittis, 1974). However, the Ranschburg effect shows that serial recall can be hindered for a repeated item (see Jahnke, 1969).

Jahnke (1972) investigated this Ranschburg effect, asking participants to learn and recall lists of seven words. Control lists contained unique words, whereas experimental lists repeated words at specific serial positions (intra-list repetition). In addition, the words used across trials were either unique or selected from a smaller pool of items, ensuring regular repetition of stimuli across trials (inter-list repetition). When the words were selected from that small pool, intra-list repetition damaged recall in comparison with non-repeated items. However, when unique words were used across trials, intra-list repetition facilitated recall in comparison with non-repeated items.

Jahnke’s (1972) second experiment further investigated the mechanisms behind this effect. It resembled the first experiment, except some participants started the procedure with the small word set, then switched to the large word set, whereas others undertook the reversed order. For those starting with the small stimulus pool, recall was typically worse for repeated than control words, but this effect was reversed when the switch to the larger word pool occurred. The opposite pattern was found for those who switched from an initial large pool to the small one, in that the facilitatory effect of within-trial word repetition was replaced by an inhibitory effect of repetition when the switch happened.

Jahnke (1972) attributed the damaging effect of repetition to PI, suggesting that it plays a critical role in the Ranschburg effect. Although non-PI explanations of the Ranschburg effect have been proposed (Henson, 1998; Hinrichs et al., 1973; Mewaldt & Hinrichs, 1977; Walsh & Schwartz, 1977), repetition of items within a sequence can be detrimental and the repeated-unique paradigm (RUP; Endress & Potter, 2014; Shoval et al., 2020) also shows that stimulus repetition impairs recognition memory.

In the RUP, participants are typically asked to encode a sequence of rapidly presented items, which are followed by a probe. Memory performance tends to be lower when stimuli are repeated throughout the procedure, rather than being unique, which is taken as evidence for PI (Endress, 2022; Endress & Potter, 2014; Endress & Siddique, 2016; Makovski, 2016). Although this effect has largely been found using visual stimuli, it has been documented for meaningful words too (Endress & Potter, 2014, Experiment 5). Given work into the Ranschburg effect and the findings from the RUP, it is plausible that experimental familiarity, as manipulated via the stimulus pool, will influence PI in the recent-probes task. The three models of this form of PI—familiarity-inhibition, context-retrieval, and biased-competition—all seem to expect repetition to increase PI. From a familiarity-inhibition perspective, repeated stimuli will have a stronger familiarity signal and increase the likelihood of match responses, which is especially damaging on RN trials. From a biased-competition view, repetition may increase the similarity between the attentional template and each probe, also increasing match responses, whereas the context-retrieval model may expect repetition to reduce the episodic details available to separate events on different trials.

Beyond the experimental setting, pre-experimental familiarity with stimuli may also be important. This relates to the role of long-term memory (LTM) and knowledge in PI (see Craik & Birtwistle, 1971), and within the wider literature, there has been some effort to try and limit the role of pre-experimental familiarity by using novel stimuli. This might include the use of random combinations of consonants in the Brown–Peterson task (Bunt & Sanders, 1972; Wickens et al., 1963), or incorporating abstract, artificially generated and entirely unfamiliar visual stimuli into the recent-probes task (McKeown et al., 2014, 2020; Mercer & Duffy, 2015). Yet the effect this has is unclear. It is plausible that an RN probe with high pre-experimental familiarity may enhance PI, as it further increases the familiarity of the RN probe (i.e., there is a detrimental combination of recency and pre-experimental familiarity). Data from the study by Shoval and Makovski (2022) are broadly consistent with this possibility, as they found that repetition increased PI, but only for meaningful stimuli. However, their data came from the RUP, which concerns non-specific PI, and the effect was found using visual material.

Conversely, pre-experimental familiarity could be advantageous as it allows the usage of stored long-term knowledge. In explaining PI in verbal memory, Tehan and Humphreys (1995) differentiated between phonological and central information. The former concerns short-lived sound- and speech-based information (e.g., rhymes), whereas the latter includes more meaningful information (e.g., the semantic nature or category membership of items), which is part of long-term knowledge. Tehan and colleagues have argued that these different types of information can influence whether PI is manifested, and there is good support for this account across multiple experiments (Tehan & Humphreys, 1995, 1996, 1998; Tolan & Tehan, 1999). It is, therefore, possible that stimuli with higher pre-experimental familiarity will be less susceptible to PI, as information about those items, including central semantic information, makes it easier to discriminate between target and interfering memories.

Unfortunately, direct assessments of pre-experimental familiarity on item-specific PI remains unclear, as studies using the recent-probes task tend to use either well-known verbal items, such as letters or words (Berman et al., 2009; D’Esposito et al., 1999; Nee et al., 2007) or abstract visual stimuli (McKeown et al., 2014, 2020; Mercer & Markova, 2023). Pre-experimental familiarity is, therefore, often confounded with stimulus type (Prabhakaran & Thompson-Schill, 2011), with some studies comparing familiar verbal items against unfamiliar visual stimuli (Badre & Wagner, 2005; Mecklinger et al., 2003). However, this issue was directly investigated by Prabhakaran and Thompson-Schill in a recent-probes task using names and faces of famous or non-famous individuals. Although there was evidence for a PI effect for all stimuli, it was larger for famous than non-famous stimuli. Prabhakaran and Thompson-Schill suggested that stimuli of famous individuals, with high pre-experimental familiarity, have richer semantic representations that heightens the familiarity signal at the time of retrieval, increasing PI. Although this would be in line with familiarity-inhibition, context-retrieval and biased-competition models, this effect only occurred in the third experiment under speeded response conditions and with face stimuli. This finding does not appear to have been replicated with other stimuli, and Prabhakaran and Thompson-Schill did not find this effect with verbal stimuli (names).

Overall, the role of familiarity in PI is complex. Although models of item-specific PI in the recent-probes task emphasise recency familiarity to explain the detrimental effect of RN probes, other forms of familiarity may increase PI from these stimuli. Potential factors include temporal familiarity (the time separating the probe from its original presentation), experimental familiarity (repetition of specific stimuli throughout the task), and pre-experimental familiarity (earlier experience with the stimuli). If these forms of familiarity do impact PI, it would suggest that there may be a role for LTM within the recent-probes task, despite this being considered a short-term/working memory procedure.

Yet as noted above, existing evidence is unclear. Reducing temporal familiarity by increasing inter-trial intervals may alleviate non-specific PI (Gorfein & Jacobson, 1972; Kincaid & Wickens, 1970; Loess & Waugh, 1967; Peterson & Gentile, 1965; Turvey et al., 1970), but it is unclear if this occurs for the item-specific PI found in the verbal recent-probes task, as relevant studies have been affected by ceiling effects. Repetition of stimuli in a PI task—which increases experimental familiarity—has yielded inconsistent effects in the wider literature (Cermak, 1969, 1970; Endress & Potter, 2014; Jahnke, 1972) and received little direct investigation in the recent-probes task, with a few exceptions (Tays et al., 2009). Finally, pre-experimental familiarity, which allows the assessment of previously formed LTM on PI, is typically confounded with the stimuli used. The one prior attempt to directly investigate pre-experimental familiarity effects on verbal PI found it had little impact (Prabhakaran & Thompson-Schill, 2011), though this may have been an artefact of the specific stimuli used (the names of famous or non-famous individuals).

As such, it remains unclear whether PI in the recent-probes task—manifested in differences between RN and NRN probes—is influenced by additional forms of familiarity beyond recency. This also creates theoretical uncertainty. In addition, no study appears to have considered the impact of multiple forms of familiarity in one experiment, but this is important as different types of familiarity may interact to further exaggerate PI. For instance, at very short inter-trial intervals, regular repetition of previously familiar stimuli may yield the strongest overall PI effect. Examining the effect of individual forms of familiarity in isolation, as is common in past work, may miss these more complex processes.

This study, therefore, investigated this issue in more depth. Participants were shown eight targets on each trial, followed by a probe. On positive trials, the probe matched one of the current targets, but on RN trials, it matched an untested target from the previous trial. On NRN trials, the probe matched an untested target from three previous trials. After responding, the next trial began after 100 ms or 10.1 s, which manipulated temporal familiarity by creating short and long inter-trial intervals. To influence experimental familiarity, targets were drawn from a repeating pool of 16 items (creating extensive repetition and high experimental familiarity) or a larger pool of unique targets (ensuring novelty and low experimental familiarity), in line with the RUP (Endress & Potter, 2014). To manipulate pre-experimental familiarity and the availability of pre-existing LTMs, stimuli were either meaningful real words (high pre-experimental familiarity) or meaningless non-words (low pre-experimental familiarity).

Performance was expected to be worse on RN than NRN trials, reflecting the typical PI effect. Familiarity-inhibition models may also expect PI to increase for real words, repeated stimuli, and short inter-trial intervals, as they all exaggerate RN familiarity. Context-retrieval models make the same prediction, as increased familiarity makes it difficult to correctly assign RN probes to a previous trial context, and the biased-competition model expects multiple forms of familiarity to increase the similarity between the attentional template and the RN probe. As such, these forms of familiarity might also interact to produce maximal PI, where repeated words presented after short inter-trial intervals should be especially detrimental. In contrast, it is possible that higher experimental and pre-experimental familiarity allow LTM to support task performance, creating a facilitatory effect that lowers PI (see Oberauer et al., 2017 for a discussion). This would be consistent with the notion that the availability of different types of information can help discriminate target memories from interfering memories (Tehan & Humphreys, 1995, 1996, 1998; Tolan & Tehan, 1999).

## Methods

### Participants

A complex experimental design was used, but of particular interest were interactions between different forms of familiarity. Brysbaert (2019) has highlighted the challenges with apriori power analysis but based on mixed 2×2 interactions with 80% power at .05 significance, Brysbaert’s suggested sample was 200 (when *d* = 0.40), 130 (when *d* = 0.50), or 90 (when *d* = 0.60). Effort was made to recruit the upper limit of 200 participants, which provides much greater power than most previous studies using the recent-probes task. However, one individual asked to withdraw their data, and a further seven were removed due to missing responses on 15%+ of trials. The final sample comprised 192 participants (*M*_age_ = 39.71, *SD* = 13.44, range = 62), including 104 women, 84 men, 2 non-binary/third gender, and 2 missing responses. The study was advertised via Prolific Academic (https://www.prolific.com/) to participants in the United Kingdom or United States, who all reported high proficiency with the English language. Participants were paid £5.50 for completing the experiment and provided their informed consent prior to undertaking the procedure. The experiment was approved by a Faculty Ethics Committee.

### Materials

Meaningful stimuli with high pre-experimental familiarity were words from the Bristol Norms database (Stadthagen-Gonzalez & Davis, 2006). Following the study by Berman et al. (2009), words were between four and six letters in length, and had one or two syllables (e.g., “grin,” “blaze,” “cement”). To create a large stimulus pool with low experimental familiarity, 928 words were selected (864 for the main experiment and 64 for practice trials). Words were randomly chosen, except offensive or highly emotional words were removed. From this larger set, 16 words were chosen for the small stimulus pool to ensure repetition and high experimental familiarity. The same 16 words were used for all participants and this number made it possible to ensure unique targets could be presented across at least two trials, while also being consistent with many previous RUP studies, which have often selected repeated items from a pool of 22 stimuli (Endress, 2022; Endress & Potter, 2014; Endress & Siddique, 2016; Shoval et al., 2020; Shoval & Makovski, 2021, 2022).

For this repeating set, a word could only be used once in a specific target sequence, and this sequence was randomly determined (words were then repeated between 52 and 68 times as targets and probes). The unique stimulus set was structured so that targets and probes on every trial matched the length of a corresponding repeated word, ensuring word length was controlled.

For stimuli with low pre-experimental familiarity, meaningless non-words were selected from the ARC non-word database (Rastle et al., 2002; http://www.cogsci.mq.edu.au/research/resources/nwdb/nwdb.html). Examples include “pite,” “broff,” and “snerbs.” All other characteristics of the non-words and methodological arrangements were equivalent to the arrangement for the words—only meaningfulness varied.

The experiment was created and run using the Gorilla Experiment Builder (www.gorilla.sc; Anwyl-Irvine et al., 2020). The procedure was completed online, but participants had to use either a desktop computer or laptop to undertake the experiment.

### Design and procedure

**Figure 1.**
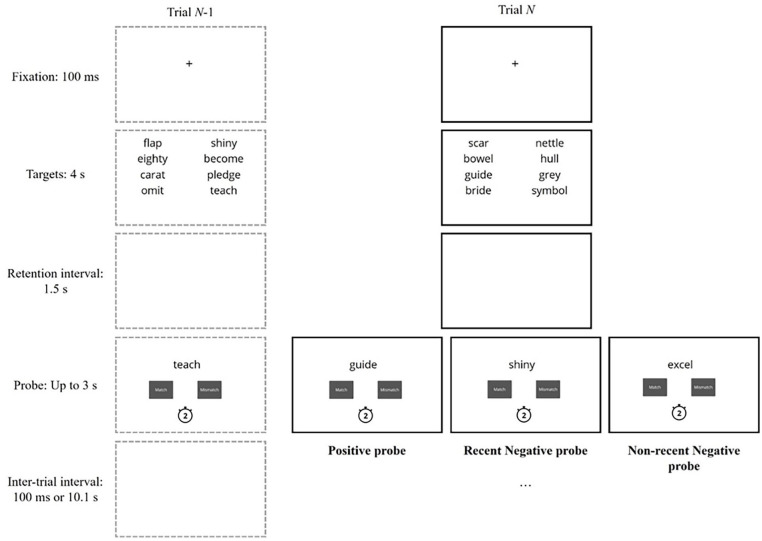
Diagram of two example trials in the recent-probes task procedure. The diagram shows two trials—the current trial is shown in black boxes and the immediately preceding trial is shown in grey boxes. The example comes from the unique condition with meaningful words.

The experimental procedure involved the recent-probes task (see Figure 1). Each trial began with a fixation cross for 100 ms followed by eight to-be-remembered target stimuli (words or non-words). Targets were presented for 4 s and organised into two columns in the upper half of the screen. Berman et al. (2009) presented four target words for 2 s, but task accuracy was extremely high. This study, therefore, doubled the number of targets, to increase difficulty, while allowing a similar encoding time for each item.

After an unfilled retention interval lasting 1.5 s, a single probe was displayed in the centre of the upper half of the screen. Two boxes appeared underneath the probe—labelled “Match” and “Mismatch”—and the task was to indicate whether the probe matched one of the current targets within 3 s. A countdown timer was presented below the response buttons to indicate the time available. The trial ended after the participant had responded or after 3 s had elapsed, and to manipulate temporal familiarity, the next trial began after 100 ms (short inter-trial interval) or 10.1 s (long inter-trial interval).

There were three probe types. Positive probes did match one of the current targets, and there were 44 positive probe types in total (22 for each inter-trial interval). These probes matched each of the eight target positions two or three times. RN probes matched an untested target from the previous trial, whereas NRN probes matched an untested target from Trial *N*-3. Other studies have also used NRN probes from Trial *N*-3 (Tays et al., 2009), showing it is sufficiently distant to provide differences between RN and NRN trials, and Berman et al. (2009) revealed that a single intervening trial can reduce PI. There were 32 occurrences of RN and NRN probes (16 per inter-trial interval) and each of the eight target positions was matched an equal number of times. Prior recent-probes task studies have used as few as 12 trials for each RN/NRN condition (Berman et al., 2009; McKeown et al., 2014, 2020), whereas this study used 16 trials to match arrangements of Mercer et al. (2022).

To make the time intervals predictable and encourage its use as a contextual feature for managing PI, trials with short and long inter-trial intervals were organised into two separate blocks. Trials within a block were initially randomised and then presented in a fixed order for participants, due to the need to control the relationship between successive trials.

Targets on experimental trials could be selected from a repeating pool of 16 items, or a unique set of 864 items, which created high and low experimental familiarity, respectively. Participants were randomly allocated to one of these sets, and randomly assigned to receive words or non-words, which was the pre-experimental familiarity manipulation.

Overall, four variables were manipulated in a mixed experimental design. Two of these were within-group manipulations—the type of probe (positive, RN, or NRN) and temporal familiarity (100 ms inter-trial interval vs 10.1 s inter-trial interval). The other two variables were manipulated between groups, including experimental familiarity (repeated vs unique targets) and pre-experimental familiarity (words or non-words).

Arrangements within the independent conditions were identical, apart from the size of the stimulus set and the type of stimuli. The order of the inter-trial interval blocks was counterbalanced, with a short break available between them, and the trials/stimuli used for the short delay for some participants was used for the long delay for other participants, and vice versa.

Prior to beginning the main experiment, participants were shown an instructional video that was succeeded by eight practice trials (four for each inter-trial interval). They then moved onto the main experimental task, which was completed without feedback. Participants were asked to respond within the 3 s time limit but without sacrificing accuracy. They were also asked to complete the study in a distraction-free environment. At the end of the procedure, participants were asked to confirm whether they were willing to submit their data for analysis or have them deleted. All participants were then given a debriefing, and the entire study lasted approximately 30 min.

### Results

The two between-groups variables—experimental familiarity and pre-experimental familiarity—created four independent conditions: repeating non-words (*N* = 54), repeating words (*N* = 47), unique non-words (*N* = 42) and unique words (*N* = 49). Mean proportion of correct responses for all probes and conditions are shown in Table 1.

**Table 1.** Mean (*SD*) proportion correct according to probe type, pre-experimental familiarity (words/non-words), experimental familiarity (repeated/unique), and temporal familiarity (100 ms or 10.1 s inter-trial interval).

	Words				Non-words			
	Repeated		Unique		Repeated		Unique	
Probe	100 ms	10.1 s	100 ms	10.1 s	100 ms	10.1 s	100 ms	10.1 s
RN	0.64 (0.23)	0.69 (0.23)	0.65 (0.21)	0.68 (0.21)	0.52 (0.20)	0.51 (0.24)	0.57 (0.22)	0.57 (0.23)
NRN	0.79 (0.17)	0.78 (0.22)	0.78 (0.20)	0.81 (0.18)	0.72 (0.18)	0.69 (0.23)	0.63 (0.22)	0.63 (0.22)
Positive	0.82 (0.12)	0.85 (0.12)	0.82 (0.13)	0.89 (0.11)	0.71 (0.14)	0.81 (0.13)	0.71 (0.15)	0.80 (0.16)

RN: recent negative; NRN: non-recent negative.

Performance on positive trials generally exceeded the negative (RN/NRN) trials and benefitted from a longer inter-trial interval—an effect not consistently observed in the RN and NRN conditions. Although positive trials are uninformative about the PI effect (see Berman et al., 2009), they can still provide useful information about task performance and so were subjected to a 2 (temporal familiarity: 100 ms inter-trial interval vs 10.1 s inter-trial interval) × 2 (experimental familiarity: repeated vs unique) × 2 (pre-experimental familiarity: words vs non-words) mixed analysis of variance (ANOVA), which met the homogeneity of variance assumption. This was supplemented with an equivalent Bayesian ANOVA, based on matched models, where BF_Inclusion_ scores are reported. This provides evidence for including a specific factor or interaction in the model (van den Bergh et al., 2020). For example, a BF_Inclusion_ score of 50 for Factor A would indicate that data are 50 times more likely for models including that factor than models that do not include it.

A significant effect of temporal familiarity did occur, *F*(1, 188) = 48.94, *p* < .001, η_p_^2^ = 0.21, and this factor had very strong evidence for inclusion (BF_Inclusion_ > 1,000,000). It was due to improved recognition when a longer delay separated trials (*M* = 0.84) compared with a shorter delay (*M* = 0.77). There was also an effect of pre-experimental familiarity, *F*(1, 188) = 32.87, *p* < .001, η_p_^2^ = 0.15, BF_Inclusion_ = 332,965.12, with better recognition of words (*M* = 0.85) than non-words (*M* = 0.76). Both main effects were qualified by an interaction between them, *F*(1, 188) = 3.88, *p* = .050, η_p_^2^ = 0.02, where the improvement in recognition at the longer delay was greater for non-words (9%) than words (5%). However, this interaction was only on the boundary of significance and had very modest evidence for inclusion (BF_Inclusion_ = 1.35). Both words and non-words benefitted from a longer inter-trial interval on positive trials.

Other effects were non-significant and lacked support (experimental familiarity: *F*[1, 188] = 0.24, *p* = .627, η_p_^2^ = 0.001, BF_Inclusion_ = 0.15; experimental familiarity × pre-experimental familiarity: *F*[1, 188] = 0.62, *p* = .433, η_p_^2^ = 0.003, BF_Inclusion_ = 0.31; temporal familiarity × experimental familiarity: *F*[1, 188] = 0.40, *p* = .529, η_p_^2^ = 0.002, BF_Inclusion_ = 0.20; temporal familiarity × experimental familiarity × pre-experimental familiarity: *F*[1, 188] = 1.88, *p* = .172, η_p_^2^ = 0.01, BF_Inclusion_ = 0.50).

When assessing performance on mismatching trials (see Table 1), there was evidence for PI, as responses on RN probes were less accurate than NRN probes in all conditions. PI was especially strong for repeated non-words, but less pronounced for unique non-words.

The PI effect was assessed using 2 (probe type: RN vs. NRN) × 2 (temporal familiarity: 100 ms inter-trial interval vs 10.1 s inter-trial interval) × 2 (experimental familiarity: repeated vs unique targets) × 2 (pre-experimental familiarity: words vs non-words) mixed frequentist and Bayesian ANOVA, which met the homogeneity of variance assumption. Four significant effects emerged, which all had strong or extremely strong support for inclusion in the model^1^. Responding was less accurate on RN (*M* = 0.61) than NRN (*M* = 0.73) trials, *F*(1, 188) = 221.97, *p* < .001, η_p_^2^ = 0.54, BF_Inclusion_ > 1,000,000, and for non-words (*M* = 0.60) than words (*M* = 0.73), *F*(1, 188) = 21.77, *p* < .001, η_p_^2^ = 0.10, BF_Inclusion_ = 1,703.02. Notably, there was little difference between repeated and unique conditions, which both averaged 0.67 correct, and this experimental familiarity variable had no support as a main effect (BF_Inclusion_ = 0.25). Yet there was a significant interaction between probe type and experimental familiarity, *F*(1, 188) = 12.68, *p* < .001, η_p_^2^ = 0.06. That interaction also received good support for inclusion (BF_Inclusion_ = 11.59), which was due to a stronger PI effect for repeated than unique conditions. However, there was also a higher order interaction between probe type, experimental familiarity, and pre-experimental familiarity that had noticeably stronger evidence for inclusion, *F*(1, 188) = 20.16, *p* < .001, η_p_^2^ = 0.10, BF_Inclusion_ = 257.58.

To examine this three-way interaction, performance on RN trials was subtracted from NRN trials to generate an estimate of PI that was collapsed across the two inter-trial intervals, as temporal familiarity had little effect. The experimental and pre-experimental familiarity variables were then treated as four independent conditions, for the purposes of breaking down the interaction. A one-way frequentist and Bayesian ANOVA was then used to compare the four conditions on the PI measure, with this analysis generating a BF^10^ score (where values of 3, 10, 30, or 100 are taken to denote moderate, strong, very strong, or extreme support for the alternative hypothesis; Wagenmakers et al., 2018). This revealed a significant effect, *F*(3, 188) = 10.71, *p* < .001, η_p_^2^ = 0.15, and extreme support for the alternative hypothesis (BF^10^ = 10,329.72). Next, a series of independent-samples *t*-tests, corrected using the Holm–Šidák adjustment (Abdi, 2010), were used to compare all four conditions. These were supplemented by Bayesian post hoc tests, which were corrected for multiple testing using an approach from the study by Westfall et al. (1997) and are also based on a BF^10^ value.

PI for unique non-words (*M* = 0.06) was smaller than repeating non-words (*M* = 0.19, *p* < .001, *d* = 1.12, BF^10^ = 33,328.30), repeating words (*M* = 0.12, *p* = .027, *d* = 0.56, BF^10^ = 4.63), and unique words (*M* = 0.13, *p* = .012, *d* = 0.67, BF^10^ = 16.12). In contrast, PI for repeating non-words was higher than both word conditions (repeating words: *p* = .010, *d* = –0.62, BF^10^ = 12.90; unique words: *p* = .036, *d* = 0.47, BF^10^ = 2.62), though from a Bayesian perspective, the latter comparison yielded only anecdotal evidence for the alternative hypothesis (Wagenmakers et al., 2018). There was no difference between repeating and unique words and this comparison was in line with the null hypothesis (*p* = .528, *d* = 0.13, BF^10^ = 0.26).

**Figure 2.**
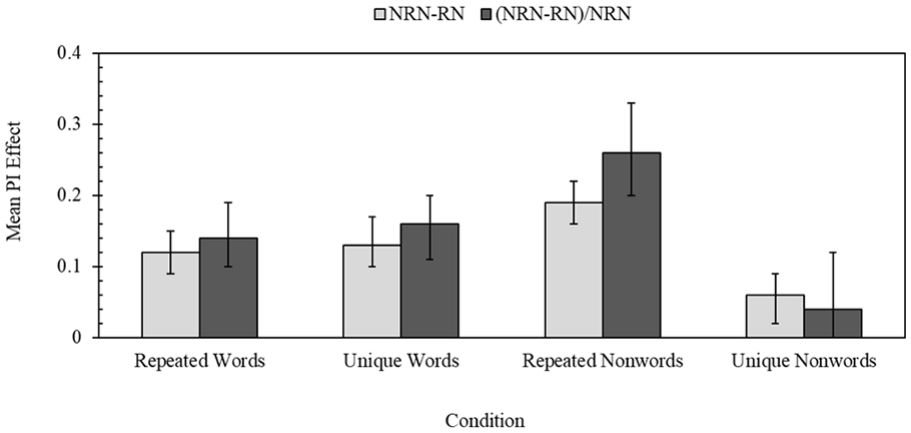
Mean PI effect according to condition based on two PI measures. Error bars show 95% CIs. Light grey columns show PI estimated by subtracting RN scores from NRN scores, whereas dark grey columns show PI estimated by subtracting RN scores from NRN scores and dividing the result by the NRN condition. In both cases, higher scores denote greater PI.

One issue with using the RN-NRN difference is that it does not account for baseline differences in performance. An alternative measure of calculating PI was therefore used, which was influenced by an approach used by Shoval and colleagues in the RUP (Shoval et al., 2020; Shoval & Makovski, 2021, 2022). This method again took NRN performance as an estimate of the upper limit of performance on mismatching trials, and subtracted RN scores from it. The result was then divided by the NRN score. This score was calculated separately for each inter-trial interval, and then the mean of the two scores used to create the final PI value. Data are shown in Figure 2, which are broadly consistent with the NRN–RN contrast.

The alternative measure of PI was also assessed with a one-way ANOVA that compared the four between-group conditions and found a significant effect, *F*(3, 186) = 8.96, *p* < .001, η_p_^2^ = 0.13, and extreme evidence for the alternative hypothesis (BF^10^ = 1,332.92). Holm–Šidák adjusted independent-samples *t*-tests and Bayesian post hoc tests were again performed, and these found PI for repeated non-words to be higher than all other conditions (unique non-words: *p* < .001, *d* = 0.90, BF^10^ = 527.79; repeated words: *p* = .025, *d* = 0.57, BF^10^ = 7.03; unique words: *p* = .036, *d* = 0.52, BF^10^ = 3.99). In contrast, unique non-words yielded the lowest amount of PI (unique words: *p* = .030, *d* = 0.56, BF^10^ = 4.47; repeated words: *p* = .047, *d* = 0.49, BF^10^ = 2.15), though the latter effect was insensitive. The two conditions using meaningful words did not differ and the effect was consistent with the null hypothesis (*p* = .696, *d* = 0.08, BF^10^ = 0.23).

Response times can also be used to assess PI, but this yielded fewer effects, though responses to RN probes were slower than NRN probes. The full analysis of those data is included in the online Supplementary Material, as the priority for this study was to assess PI on an accuracy measure when ceiling effects were avoided.

## Discussion

This study examined how PI in the verbal recent-probes task—a recency-based effect in which RN probes impair performance more than NRN probes—could be influenced by different forms of familiarity. This was motivated by theoretical uncertainty concerning the impact of other forms of familiarity beyond simple recency and mixed empirical evidence.

A clear PI effect was found, being manifested in more error-prone performance on RN than NRN trials (and slower responding—see the online Supplementary Material). This supports numerous prior studies using the recent-probes task (Badre & Wagner, 2005; Berman et al., 2009; Campoy, 2012; D’Esposito et al., 1999; McKeown et al., 2014, 2020; Mecklinger et al., 2003; Mercer & Duffy, 2015; Nee et al., 2007; Tays et al., 2009) and is consistent with the detrimental effect of PI on verbal memory. However, the major novel contribution of this study was the discovery that different forms of familiarity interact to influence PI, but in a way that was problematic for some current models of PI and some previous empirical findings.

Based on existing theories of item-specific PI—familiarity-inhibition, context-retrieval and biased-competition—it was expected that temporal, experimental, and pre-experimental familiarity would all influence the magnitude of PI. Although these accounts have primarily been applied to the RN/NRN difference, other familiarity-based manipulations were predicted to affect the impact of RN probes. For familiarity-inhibition, short inter-trial intervals (high temporal familiarity), repeating stimuli (high experimental familiarity), and meaningful words (high pre-experimental familiarity) might make the incorrect endorsement of RN probes even more likely, strengthening PI. For context-inhibition, this heightened familiarity would increase the overlap between different trial contexts, making it more challenging to reject an RN probe by assigning it to an earlier trial context, and with biased-competition, the attentional template used to complete the task may become more similar to the RN probe as familiarity increases.

The results, however, were not fully consistent with these ideas. Experimental familiarity, induced by stimulus repetition, was insufficient in isolation to increase PI. A main effect of experimental familiarity was not found on either mismatching or matching trials, which is inconsistent with some past work. Tays et al. (2009), for example, found an increase in errors in the recent-probes task when stimuli were selected from a smaller rather than a larger pool of words. However, this study did not find an interaction between the type of probe and the degree of stimulus repetition, and the error rate on NRN trials was low (< 4%).

The absent main effect of experiment familiarity also conflicts with studies using the RUP, in which repetition of stimuli tends to lower performance compared with unique stimuli (Endress, 2022; Endress & Potter, 2014; Endress & Siddique, 2016). However, repetition was detrimental when non-words were used, hence there was a crucial interaction between experimental and pre-experimental familiarity. This was not in the manner expected by the three models of PI outlined above, which predicted repeated words to produce the strongest PI. Instead, PI for repeating non-words was stronger than all other conditions, whereas unique non-words led to the smallest PI effect. When meaningful words were targets, robust PI was found for both repeated and unique stimuli, and repetition did not significantly increase PI.

These data, therefore, showed more complex effects of pre-experimental familiarity than initially predicted. Based on some previous evidence, it was hypothesised that stimuli with high pre-experimental familiarity would increase PI as these items link to stored semantic knowledge and have richer representations (Prabhakaran & Thompson-Schill, 2011; Shoval & Makovski, 2022), which increase the familiarity of RN probes. Such an effect was predicted by familiarity-inhibition and biased-attention models, but high pre-experimental familiarity could also reduce the availability of contextual information that can separate different events in memory, as predicted by the context-retrieval account.

The current findings were inconsistent with this possibility, challenging the three models of PI. The present data also conflicted with both Prabhakaran and Thompson-Schill (2011), who found higher pre-experimental familiarity (famous individuals) increased PI in the recent-probes task, and Shoval and Makovski (2022), who found that repeated stimuli led to lower performance than unique stimuli, but only for meaningful stimuli. However, the effect of the study by Prabhakaran and Thomspon-Schill was somewhat limited. In the first two studies, four targets—names or faces, in separate blocks—were presented simultaneously within a recent-probes task. This did not reveal any effect of pre-experimental familiarity on the behavioural PI measure, which only occurred in a subsequent experiment using face stimuli under speeded response times. The equivalent effect was not found with verbal stimuli, but as these were names, they still had some meaningfulness. In contrast, Shoval and Makovski (2022) used genuinely meaningless stimuli—photos of everyday objects that had been scrambled to become heavily distorted—and found repetition only enhanced PI for meaningful items. This is notably different to the present finding, but there are key differences between their study and the current experiment, particularly in terms of the stimuli and the PI manipulation. There is also a potential artefact within the RUP used by Shoval and Makovski (2022), in that the repeated condition selects both targets and probes from a limited set of items, whereas in the unique condition, probes are also novel. The disadvantage typically reported in the repeated condition may, therefore, actually occur because it is easy to reject entirely novel probes, not previously encoded, in the unique condition. Tentative evidence for this possibility is seen in the false alarm rate data of previous RUP studies (Endress & Potter, 2014; Endress & Siddique, 2016; Shoval & Makovski, 2021). In contrast, this study ensured that all mismatching probes had previously been encountered, with the only difference being in terms of the recency of this encounter.

Although the novel interaction between experimental and pre-experimental familiarity is inconsistent with some prior studies and the familiarity-inhibition, context-retrieval, and biased-attention models, it can be explained with reference to other PI work. As noted above, Tehan and Humphreys (1995) have argued that different components of verbal traces can be used to manage PI, including semantic/central information. Non-word stimuli lack this element, but it only becomes critical for PI when these stimuli are extensively repeated throughout the task. The repetition of non-words is likely to have created new LTMs, and there is a well-established idea that PI is a component of long-term or secondary memory (Craik & Birtwistle, 1971). Ordinarily, meaningless non-words would not be expected to connect to pre-existing semantic knowledge (see Coltheart & Geffen, 1970), explaining why PI was more modest for unique non-words. That is, limited PI for unique non-words may have been because these stimuli were held in a transient working memory buffer, and thereby performance reflected a genuinely short-term effect. However, with repeated repetition, durable memories for the non-word stimuli should have developed, yet an absence of prior semantic representations may have made it difficult to discriminate between old and new events in memory, resulting in heightened PI.

The same effect did not occur for meaningful words, where there was no difference between repeated and unique conditions. Again, however, this can be explained with reference to the role of LTM and semantic representations. Participants may have used semantic information about the meaningful words to prevent PI from becoming too intense when stimuli were frequently repeated. Although it was not possible to fully overcome PI for repeated words, PI did not reach the levels recorded for repeated non-words, suggesting that long-term knowledge about the words was used as an additional cue to segregate information across trials. That is, instead of contextual overlap always increasing PI, as predicted by context-retrieval accounts, it may have been used to prevent PI from becoming too extreme when meaningful stimuli were regularly repeated. This is consistent with the model of PI by Tehan and Humphrey (1995), in which central information about the stimuli is one cue for managing PI.

Further support for a role of LTM and stored knowledge comes from the limited effect of temporal familiarity. Although the gap separating trials could be very short (100 ms) or much longer (10.1 s), PI was present at both delays, highlighting LTM contributions. Supporting this, some studies using the Brown–Peterson task have shown that temporal delays between trials need to be lengthy—well beyond the typical duration of short-term memory—before clear reductions in PI are reported (Kincaid & Wickens, 1970). The absence of a temporal familiarity effect is also consistent with the work of Berman et al. (2009), though importantly this study was able to document this effect on a proportion correct measure, having avoided ceiling effects by employing a greater number of stimuli on each trial. It should also be noted that extending the inter-trial interval did have a beneficial effect on the positive trials, suggesting that temporal familiarity may still be an important factor for retention outside the PI effect.

Overall, this study has provided new insights into how different forms of familiarity can affect PI, though there were some limitations. The probe type, experimental, and pre-experimental familiarity interaction was only revealed on the accuracy measure, though this was of particular importance given prior concerns about ceiling effects. It is also not uncommon for PI to behave differently on accuracy and response time measures. For example, when task accuracy is extremely high, PI may only manifest in response times (D’Esposito et al., 1999), whereas in other recent probes-task studies, the PI effect has manifested only in the accuracy data (McKeown et al., 2020, Experiments 2–5). As already noted, this study was especially motivated to examine the error rate and participants were asked to respond within the time limit, but without sacrificing accuracy, placing greater emphasis on correct responding. This may explain why the familiarity influence on PI was more clearly revealed in proportion correct data.

Future studies could explore this further, perhaps manipulating the type of instruction given (whether to emphasise response speed or accurate responding), which prior work had indicated is important (Prabhakaran & Thompson-Schill, 2011). It would also be useful to adapt the current design for use with visual stimuli, to assess whether the effects of familiarity do respond differently when meaningful and meaningless images are used within the recent-probes task.

Another potential limitation was the wide age range of participants who were recruited, as past work has shown that PI can be stronger in older adults (Samrani et al., 2017). However, although this study allowed anyone aged 18 or over to volunteer, only eight individuals were aged over 65. There was a marginal correlation between age and the PI measure based on the NRN–RN contrast (*r* = 0.13, *p* = .074), with older adults experiencing greater PI, but a Bayesian correlation suggested this relationship was more in line with the null hypothesis, though insensitive (BF^10^ = 0.44). In addition, age did not significantly correlate with the PI measure based on the estimate by Shoval and colleagues ([NRN-RN]/NRN; Shoval et al., 2020; Shoval & Makovski, 2021, 2022; *r* = 0.08, *p* = .262) and this correlation was consistent with the null hypothesis (BF^10^ = 0.17). Finally, mean age of the participants in the four conditions was extremely similar, varying between 38.0 and 40.9, with a Bayesian ANOVA testing whether age differed according to condition revealing convincing evidence for the null hypothesis (BF^10^ = 0.04).

In conclusion, the effect of PI on verbal memory depends on the wider familiarity of the stimuli that are used. In particular, a critical interaction between experimental familiarity and pre-experimental familiarity was revealed, in which PI was strongest for repeated non-words, and smallest for unique non-words. Yet stimulus repetition had little effect for meaningful words and temporal familiarity was unrelated to PI. The present findings revealed more complex effects of familiarity than have previously been assumed and support a role for LTM in verbal PI. To understand how stimuli from the recent past disrupt current processing, it is necessary to consider dual forms of familiarity, including repetition of stimuli within the experimental procedure, and prior familiarity with stimuli outside the experimental setting.

## Supplemental Material

sj-docx-1-qjp-10.1177_17470218251317191 – Supplemental material for Familiarity influences on proactive interference in verbal memory Supplemental material, sj-docx-1-qjp-10.1177_17470218251317191 for Familiarity influences on proactive interference in verbal memory by Tom Mercer in Quarterly Journal of Experimental Psychology

## Notes

1. Temporal familiarity was non-significant and unsupported, *F*(1, 188) = 0.60, *p* = .439, η_p_^2^ = 0.003, BF_Inclusion_ = 0.18, as was its interaction with experimental familiarity, *F*(1, 188) = 0.81, *p* = .369, η_p_^2^ = 0.004, BF_Inclusion_ = 0.11, and its interaction with pre-experimental familiarity, *F*(1, 188) = 2.76, *p* = .098, η_p_^2^ = 0.01, BF_Inclusion_ = 0.82. The three-way interaction between temporal, experimental, and pre-experimental familiarity was non-significant too and had no justification for inclusion, *F*(1, 188) = 0.13, *p* = .721, η_p_^2^ = 0.001, BF_Inclusion_ = 0.13. The interaction between probe type and pre-experimental familiarity, *F*(1, 188) = 0.04, *p* = .851, η_p_^2^ < 0.001, BF_Inclusion_ = 0.10, probe type and temporal familiarity, *F*(1, 188) = 1.25, *p* = .265, η_p_^2^ = 0.01, BF_Inclusion_ = 0.58, probe type, temporal familiarity, and experimental familiarity, *F*(1, 188) = 1.50, *p* = .222, η_p_^2^ = 0.01, BF_Inclusion_ = 0.64, probe type, temporal familiarity, and pre-experimental familiarity, *F*(1, 188) = 0.85, *p* = .357, η_p_^2^ = 0.01, BF_Inclusion_ = 0.05, and the interaction between all four variables, *F*(1, 188) = 0.55, *p* = .459, η_p_^2^ = 0.003, BF_Inclusion_ = 0.07, were non-significant and had no evidence for inclusion either. There was little effect of experimental familiarity, *F*(1, 188) = 0.002, *p* = .966, η_p_^2^ < 0.001, BF_Inclusion_ = 0.25, and no interaction between experimental and pre-experimental familiarity, *F*(1, 188) = 0.08, *p* = .781, η_p_^2^ < 0.001, BF_Inclusion_ = 0.26.
