# Supplementary material for: Familiarity influences on proactive interference in verbal memory
Source: Q J Exp Psychol (Hove). 2025 Feb 14;78(9):2008–21. doi: 10.1177/17470218251317191 (PMC12335633; doi:10.1177/17470218251317191)
Supplement: sj-docx-1-qjp-10.1177_17470218251317191 – Supplemental material for Familiarity influences on proactive interference in verbal memory [file sj-docx-1-qjp-10.1177_17470218251317191.docx]

Supplementary Material for:

Familiarity Influences on Proactive Interference in Verbal Memory

Tom Mercer

School of Psychology

University of Wolverhampton

Tom Mercer <https://orcid.org/0000-0001-8402-1762>

Correspondence concerning this article should be addressed to Tom Mercer, Centre for Psychological Research, School of Psychology, University of Wolverhampton, Wolverhampton, UK, WV1 1LY. Email: [t.mercer2@wlv.ac.uk](mailto:t.mercer2@wlv.ac.uk). Telephone: +44(0)1902 321368.

The data that support the findings of this study are openly available in the Open Science Framework project “Familiarity Influences on Proactive Interference in Verbal Memory” at <https://osf.io/f42ay/>

**Table 2**

*Mean (SD) Response Time (ms) According to Probe Type, Pre-experimental Familiarity (Words/Nonwords), Experimental Familiarity (Repeated/Unique) and Temporal Familiarity (100 ms or 10.1 s Inter-trial Interval)*

|  | Words | | | |  | Nonwords | | | |
| --- | --- | --- | --- | --- | --- | --- | --- | --- | --- |
|  | Repeated | | Unique | |  | Repeated | | Unique | |
| Probe | 100 ms | 10.1 s | 100 ms | 10.1 s |  | 100 ms | 10.1 s | 100 ms | 10.1 s |
| RN | 1518.62 (299.90) | 1738.16 (348.96) | 1395.45 (229.49) | 1555.17 (255.48) |  | 1454.61 (268.97) | 1681.19 (320.07) | 1425.29 (279.16) | 1652.14 (331.34) |
| NRN | 1454.87 (270.54) | 1651.43 (314.92) | 1312.71 (189.03) | 1504.55 (260.77) |  | 1414.30 (248.05) | 1626.48 (354.71) | 1414.46 (277.50) | 1614.91 (314.70) |
| Positive | 1300.14 (236.43) | 1499.54 (251.02) | 1205.10 (211.96) | 1376.38 (220.42) |  | 1322.92 (221.99) | 1470.49 (250.13) | 1302.99 (272.86) | 1548.94 (324.75) |

In some previous studies using the recent-probes task, response time data has been assessed with errors removed (e.g., Berman et al., 2009; Mercer et al., 2022; McKeown et al., 2020). The present task was designed to be challenging, however, revealing a higher error rate than is common in the recent-probes task, especially for verbal stimuli. Yet analysing response time data with errors included did not reveal any different effects to the analysis with errors removed, with a few exceptions that are explained below, so the latter is included here for consistency with past work.

The mean response times, with errors removed, are shown in Table 2. A PI effect was revealed, with responding on RN trials being slower than NRN trials in every condition, whereas responding was most rapid on Positive trials. Responding also consistently slowed down at the longer inter-trial interval.

The analysis on Positive trials found a significant effect of temporal familiarity, *F*(1, 188) = 192.05, *p* < .001, η_p_^2^ = 0.51, BF_Inclusion_ > 1000000, due to slower responding after the 10.1 s inter-trial interval (*M* = 1471.23 ms) than the 100 ms inter-trial interval (*M* = 1279.05). There was also a significant interaction between experimental and pre-experimental familiarity, *F*(1, 188) = 4.77, *p* = .030, η_p_^2^ = 0.03, though evidence for this was ambiguous (BF_Inclusion_ = 1.99). All three variables also significantly interacted, *F*(1, 188) = 4.81, *p* = .030, η_p_^2^ = 0.03, though again the Bayesian analysis suggested limited evidence for this interaction (BF_Inclusion_ = 1.86), and the overall model was dominated by the effect of temporal familiarity. Other effects were non-significant and unsupported: Experimental familiarity: *F*(1, 188) = 0.98, *p* = .324, η_p_^2^ = 0.01, BF_Inclusion_ = 0.40; pre-experimental familiarity: *F*(1, 188) = 2.93, *p* = .089, η_p_^2^ = 0.02, BF_Inclusion_ = 0.75; temporal familiarity x experimental familiarity: *F*(1, 188) = 1.58, *p* = .211, η_p_^2^ = 0.01, BF_Inclusion_ = 0.32; temporal familiarity x pre-experimental familiarity: *F*(1, 188) = 0.17, *p* = .678, η_p_^2^ = 0.001, BF_Inclusion_ = 0.16. The analysis including errors revealed the same pattern of results, except the interaction between experimental and pre-experimental familiarity became only marginally significant (*p* = .06).

To assess PI, a 2 (probe type: RN vs. NRN) x 2 (temporal familiarity: 0.1 s inter-trial interval vs. 10.1 s inter-trial interval) x 2 (experimental familiarity: repeated vs. unique) x 2 (pre-experimental familiarity: words vs. nonwords) mixed ANOVA, with an equivalent Bayesian ANOVA, was also performed. Participants were significantly slower responding to RN (*M* = 1552.58 ms) than NRN (*M* = 1499.21) probes, in line with the typical PI effect. They were also significantly slower to respond after a longer inter-trial interval (100 ms: 1423.79 ms, 10.1 s: *M* = 1628.00 ms), and for repeated (*M* = 1567.46 ms) than unique (*M* = 1484.34 ms) stimuli. No other effects or interactions were significant, though interactions between probe type and pre-experimental familiarity and between experimental familiarity and pre-experimental familiarity were marginal. The former interaction was due to a more pronounced PI effect for words than nonwords (RN trials being 71 ms slower than NRN trials for words, but only 36 ms slower for nonwords). However, this had limited evidence for inclusion (BF_Inclusion_ = 0.29). The latter interaction, between experimental and pre-experimental familiarity, occurred because responding to repeated words was slower than repeated nonwords, whereas the reversed pattern was observed for unique words. This also had some evidence for inclusion from the Bayesian analysis (BF_Inclusion_ = 6.28). The full ANOVA outcomes are shown in Table 3.

The same analysis with all responses included still found significant effects of probe type and temporal familiarity, but the main effect of experimental familiarity became marginal, and no interactions were close to significance.

**Table 3**

*Output from ANOVAs on Response Time Data for Mismatch Trials*

| Effect/Interaction | *F* | *p* | η_p_^2^ | BF_Inclusion_ |
| --- | --- | --- | --- | --- |
| Probe Type | 35.24 | <.001 | 0.16 | 225190.66 |
| Temporal Familiarity | 142.47 | <.001 | 0.44 | >1000000 |
| Experimental Familiarity | 5.27 | .023 | 0.03 | 0.88 |
| Pre-experimental Familiarity | 0.28 | .599 | 0.001 | 0.37 |
| Probe Type x Temporal Familiarity | 0.18 | .673 | 0.001 | 0.08 |
| Probe Type x Experimental Familiarity | 0.79 | .374 | 0.004 | 0.10 |
| Probe Type x Pre-experimental Familiarity | 3.83 | .052 | 0.02 | 0.29 |
| Temporal Familiarity x Experimental Familiarity | 0.31 | .579 | 0.002 | 0.11 |
| Temporal Familiarity x Pre-experimental Familiarity | 0.52 | .473 | 0.003 | 0.19 |
| Experimental Familiarity x Pre-experimental Familiarity | 3.29 | .071 | 0.02 | 6.28 |
| Probe Type x Temporal Familiarity x Experimental Familiarity | 0.33 | .567 | 0.002 | 0.22 |
| Probe Type x Temporal Familiarity x Pre-experimental Familiarity | 0.44 | .507 | 0.002 | 0.15 |
| Probe Type x Experimental Familiarity x Pre-experimental Familiarity | 0.17 | .679 | 0.001 | 0.22 |
| Temporal Familiarity x Experimental Familiarity x Pre-experimental Familiarity | 0.15 | .699 | 0.001 | 0.42 |
| Probe Type x Temporal Familiarity x Experimental Familiarity x Pre-experimental Familiarity | 0.80 | .373 | 0.004 | 0.35 |
